# Experimental Investigation of Self-Assembled Particles on Profile Control in the Fuyu Oilfield

**DOI:** 10.3389/fchem.2021.681846

**Published:** 2021-08-05

**Authors:** Lifei Dong, Miao Wang, Jie He, Mingchen Ding, Hun Lin

**Affiliations:** ^1^ Chongqing Three Gorges University, Chongqing, China; ^2^ Chongqing Engineering Research Center of Disaster Prevention & Control for Banks and Structures in Three Gorges Reservoir Area, Chongqing, China; ^3^ Southwest Petroleum University, Chengdu, China; ^4^ China University of Petroleum, Qingdao, China; ^5^ Chongqing University of Science and Technology, Chongqing, China

**Keywords:** profile control, self-assembled particle, oil restarting pressure, plugging strength, fuyu oilfield

## Abstract

The particle system is one of the widely used profile control agents in many oilfields, and the matching relationship between the particle and the reservoir pore throat is significant for the profile control effect. In order to enhance oil recovery after water breakthrough in the Fuyu oilfield, a self-assembled particle with some branches on the surface, compounded by inverse emulsion polymerization and added, is introduced as the profile control agent in this paper. Then the permeabilities of the water channel and the oil remaining area in the Fuyu oilfield are achieved after the statistic analysis of 1,022 cores from the practical reservoir. Furthermore, the oil restarting pressure in the oil remaining area and the self-assembled particle plugging strength in the water channel are tested. Finally, the adaption of the self-assembled particle and effect of profile control in the Fuyu oilfield are evaluated by comparing the oil restarting pressure and the plugging strength. The results show that the self-assembled particles can be gathered together easily by the force of the ionic bond, which is good for water channel plugging. The permeability of the water channel in the Fuyu oilfield ranges from 1,000 mD to 1,500 mD. The oil restarting pressure increases with the decreasing of permeability, and the increasing rate grows rapidly when it drops below 50 mD. Comparing the oil restarting pressure with the plugging strength, a self-assembled particle with a diameter of 20–40 μm in the water channel with a permeability of 1,265.7 mD can provide sufficient plugging strength to restart the remaining oil in the oil remaining areas with a permeability over 3.38 mD. The matched window of the self-assembled particle is wider than a normal particle in the Fuyu oilfield.

## Introduction

High water cut and low oil recovery efficiency are common phenomena in most oilfields now after long-term water flooding (Feng, et al., 2013). The effective way to enhance oil recovery is to use a suitable profile control agent to plug the water channel, which exists in the high permeability areas of the reservoir. After the water channel is plugged by the agent, the subsequent injected fluid can be swept to the low permeability areas and avoid flowing along the water channel. Thus, the residual oil there can be displaced (Yadav and Mahto, 2014).

Many chemical particle systems, such as the preformed gel particle (PGP), microgels, and dispersed particle gels (DPG), are proposed by researchers as profile control agents, and the mechanism of the plugging and profile control are studied (Dai, et al., 2012; Goudarzi, et al., 2013; You, et al., 2013; Zhou, et al., 2013; Yue, et al., 2006). Bai observed elastic deformability during the migration and plugging process of the preformed gel particle in visible etched-glass micromodels, and concluded profile control mechanism and sweep efficiency improvement in the reservoir (Bai, 2001; Bai et al., 2007a; Bai et al., 2007b). Iscan, A.G. and Civan, F. showed that particles with sizes comparable to pore throat sizes can reduce the permeability effectively in high permeability formations (Iscan and Civan, 2006; Iscan, et al., 2007; Tran, et al., 2009). Al-Ibadi, A. and Civan, F. explored the effects of flow rate, concentration, and size on the plugging ability when injecting the deformable gel particles in the near-wellbore formation (Al-lbadi, et al., 2012; Al-lbadi and Civan, 2013). Zhao investigated the mechanisms of profile control and enhanced oil recovery of DPG particles with core flow tests and visual simulation experiments, and the deformation, retention, adsorption, trapping, and bridging during DPG particle migration in porous media were demonstrated (Zhao, et al., 2014).

These studies mentioned above all concentrated on the plugging ability and the matchable degree between the reservoir and the different particles. However, the restarting pressure of remaining oil in the lower permeability areas is little considered, which was exactly related to the final profile control effect and enhanced oil recovery.

As the water channel in the Fuyu oilfield has had long-term water flooding (Liu, et al., 2009), the measure of profile control should be taken urgently (Li, et al., 2010; Shu, et al., 2013).

In this work, the physical properties (permeability distribution) of 1,022 cores from the practical reservoir in the Fuyu oilfield are analyzed first. Then, a self-assembled particle with some branches on the surface is introduced as the profile control agent. The particle is compounded with the method of inverse emulsion polymerization. After that, two kind of experiments, the remaining oil restarting pressure test and particle plugging strength analysis, are designed and completed according to the theoretical foundation of profile control. Furthermore, the plugging ability of the self-assembled particle and the matchable degree between the particle and the water channel in the Fuyu oilfield is discussed. Finally, the adaption of the self-assembled particle for profile control in the Fuyu oilfield is evaluated by comparing the plugging strength with the remaining oil restarting pressure.

## Materials

### Self-Assembled Particle

The profile control agent used in the reservoir is the self-assembled particle, which is made with the method of emulsion polymerization. The main synthesis materials of the particle are styrene (St), acrylamide (AAm), and N,N′- methylenebisacrylamide (MBA).

**FIGURE 1:**
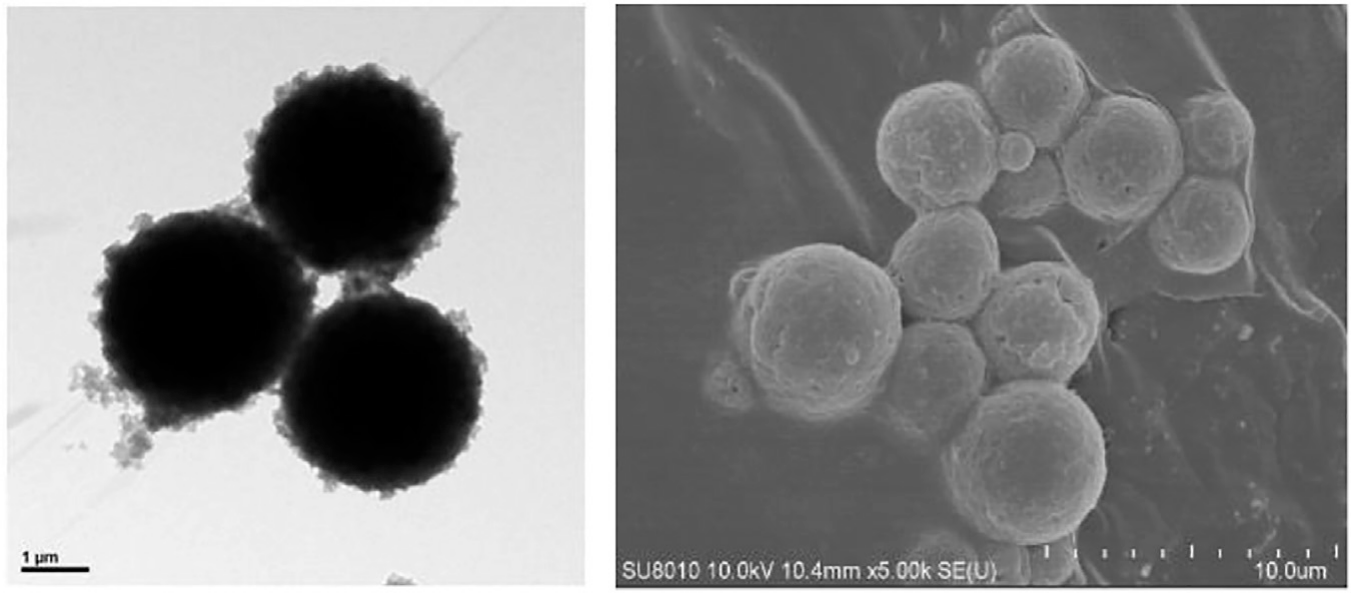
The property of self-assembly in static and dynamic conditions.

The self-assembled property of this particle is determined by its structure, which can be seen from the SEM tests in the continuous medium or in the porous media (Figure 1). This property can make single particles gather together, which is good for plugging the water channel.

### Experimental Cores

**FIGURE 2:**
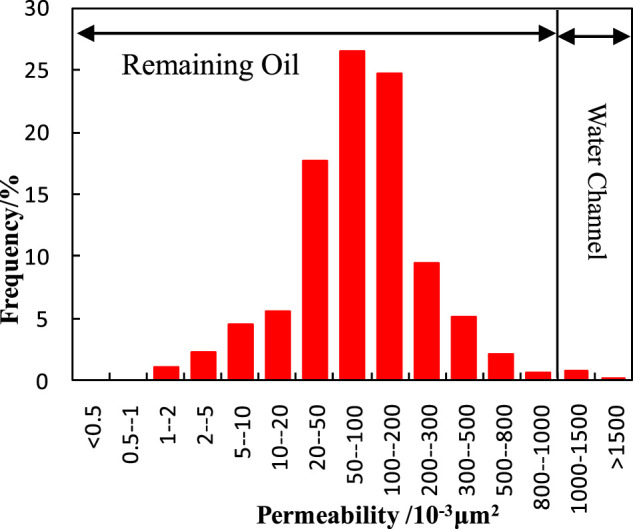
The statistical analysis of the permeability distribution.

The experimental cores are selected and confirmed after the statistical analysis of the permeability from 1,022 natural cores in the Fuyu oilfield (Figure 2).

It can be seen that the distribution frequency of permeability is normal. As the water channel exists in high permeability reservoirs, the experimental cores with the permeability above 1,000 mD are selected in this paper according to the statistical analysis.

### Experimental Fluids

The water used in the experiments is formation water, with a total salinity of 3260 mg/L. The component content of ions is shown in Table 1.

**TABLE 1:** The component content of ions in experimental water.

Ions	K^+^+Na^+^	Ca^2+^	Mg^2+^	Cl^-^	SO_4_^2-^	HCO_3_^−^	CO_3_^2-^
Content (ppm)	1,070	20	27	1,152	228	763	0

The oil is a mixture composed of gas-free crude oil and kerosene. Oil viscosity is 7.36 mPa·s at 32 °C.

## Experiments

### Theoretical Foundation

**FIGURE 3:**
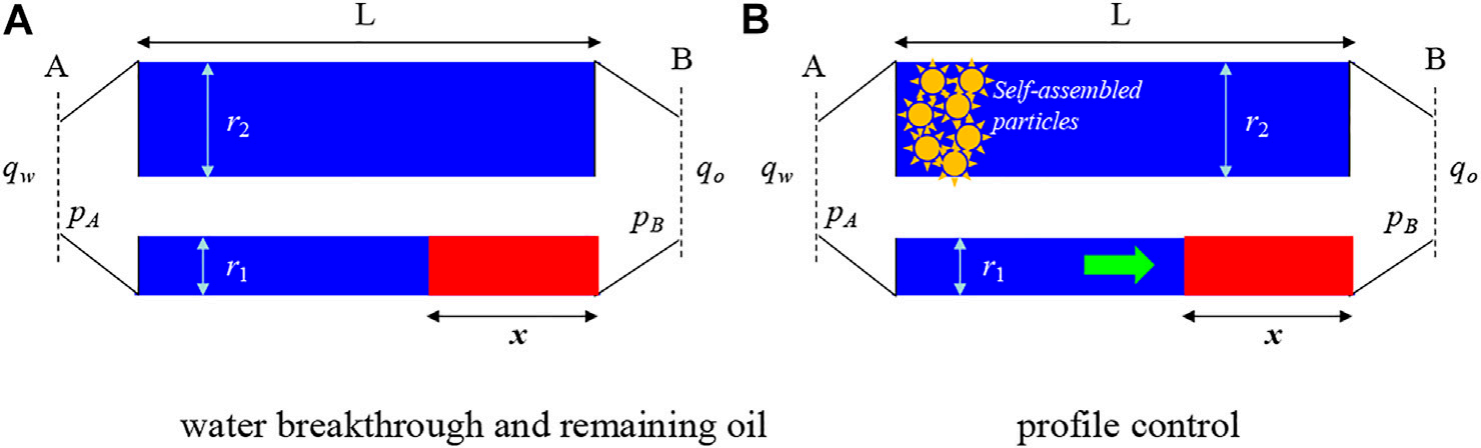
Water breakthrough and profile control.

The measure of profile control in the oilfield is taken for reducing water channeling and enhancing oil recovery. According to the research of Dong et al., 2021, water breakthrough will happen if microheterogeneity exists in the reservoir, and the oil will remain local in the high permeability areas (Figure 3A). As seen from Figure 2, the heterogeneity of the reservoir in the Fuyu oilfield is common. Thus, water breakthrough is unavoidable. The oil saturation in the smaller size capillary (or lower permeability areas) is

$$S_{o}=\frac{\mu_{o}}{\mu_{o}-\mu_{w}}\left\{\sqrt{1-{\left(\frac{r_{1}}{r_{2}}\right)}^{2}\left[1-{\left(\frac{\mu_{w}}{\mu_{o}}\right)}^{2}\right]}-\left(\frac{\mu_{w}}{\mu_{o}}\right)\right\}$$ (1)

where *S*_o_ is oil saturation; *μ*_o_, *μ*_w_ are the viscosity of oil and water; *r*_1_, *r*_2_ are the sizes of the smaller capillary (lower permeability areas) and the bigger capillary (higher permeability areas). The theoretical model can be found in Appendix A.

The self-assembled particle is used to plug the water channel (bigger-sized capillary), and the finally purpose is to restart the remaining oil in the smaller-sized capillary (low permeability areas). It means that the plugging strength of the particle in high permeability areas (*P*_r2_) should be larger than the restart pressure of oil in the low permeability areas (*P*_r1_).

As seen from Figure 3B, the condition below should be satisfied:

$$P_{r2}\geq P_{r1}$$ (2)

That is the theoretical foundation of profile control.

### Experimental Scheme

Based on the theoretical analysis, two pressures should be achieved and compared before the adaptation evaluation. So two kind of experiments are designed.

1) The oil restarting pressure experiment.

Seven different permeability cores saturated the oil are taken, and the water is injected in the cores to displace the oil. The injecting pressure is tested when the first oil drop flows out. This pressure can be represented as the oil restarting pressure. And the experimental temperature is 32°C. The basic parameters of experimental cores are shown in Table 2.

2) The plugging strength experiment of the self-assembled particle.

**TABLE 2:** Basic parameters of experimental cores.

Number	Diameter/cm	Length/cm	Oil saturation/%	Permeability/mD
H1	2.50	30.00	60.26	0.38
H2	—	—	62.53	6.88
H3	—	—	64.02	35.66
H4	—	—	64.72	56.36
H5	—	—	65.15	104.25
H6	—	—	68.87	540.12
H7	—	—	70.88	1,265.70

Two high permeability cores are used to inject the self-assembled particles. The particles will plug and migrate in the pore throat of the core, and the injecting pressure will remain stable when the plugging and migration is balanced. After that, the water injection follows. And the stable water injection pressure can be seen as the particle plugging strength.

As the particle plugging is formed in the water channel (high permeability areas), two cores with the permeability of 1,265.7 mD and 3,014.7 mD are used for the core experiments, and their basic parameters are listed in Table 3.

**TABLE 3:** Basic parameters of experimental cores.

Number	Diameter/cm	Length/cm	Permeability/mD	Porosity/%
ZR-1	2.50	30.00	1,265.70	33.40
ZR-2	2.50	30.00	3,014.70	38.10

**FIGURE 4:**
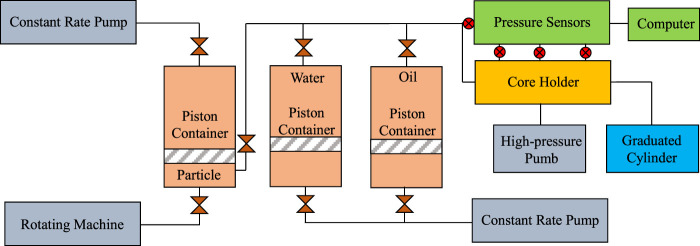
The experimental devices.

Thus, the effect of profile control in the Fuyu oilfield can be evaluated according to these two pressures. The devices of the experiments are shown below Figure 4.

## Results and Discussion

1) The oil restarting pressure in different permeability cores

**FIGURE 5:**
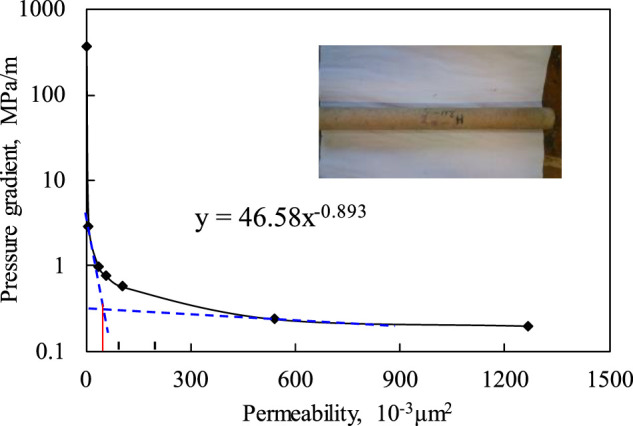
The pressure gradient of oil restarting with different permeabilities.

The pressures (or pressure gradients) tested from the experiments of different permeability cores are shown in Figure 5.

It can be seen that the oil restarting pressure increases with the decreasing of permeability. And the increasing rate grows rapidly when the permeability drops below 50 mD. That means the oil remaining in the lower permeability areas needs more driving force for restarting.

2) The plugging strength of the self-assembled particle in high permeability cores

**FIGURE 6:**
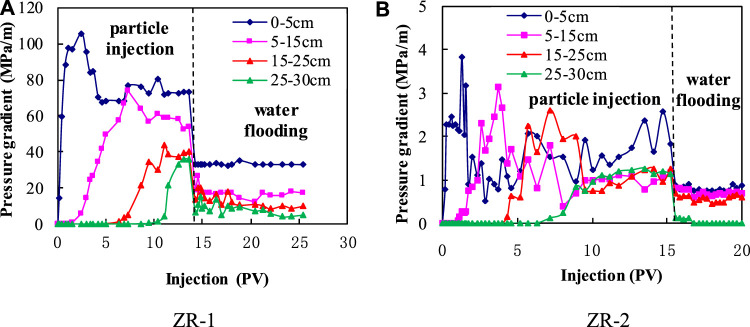
Injection dynamics of the self-assembled particle in experimental cores.

The self-assembled particle with the diameter of 20∼40 μm is used for the experiments. The concentration of the particle solution is 1,000 ppm. The particle injection rate is 1 m/d. The dynamic behaviors of pressure gradients at different position of the cores are show in Figure 6.

It can be seen that the pressure gradient at different positions rises gradually with the increasing particle injection. That means the self-assembled particle can migrate to deep within the cores. Meanwhile, the pressure gradient remains stable after injecting the particle for a while. That is because the particle retention in the core reaches its maximum. The final pressure gradient of subsequent water flooding can be represented as the plugging strength of the self-assembled particle during the profile control measure.

According to the injection dynamics of Core ZR-1, the plugging strength at 0–5 cm is much higher than the one at other positions. That is because the particle retention in the entrance is higher than that at other places, which is called the entrance face effect. The plugging strength in the center of the cores can be represented as the actual particle plugging strength. Thus, the plugging strength of the self-assembled particle in Core ZR-1 is about 14.05 MPa/m, which is achieved from the average value of point 5–15 cm and point 15–25 cm. Also, the plugging strength in Core ZR-2 is 0.64 MPa/m.

3) The evaluation of the self-assembled particle on profile control in the Fuyu oilfield

The permeability of where the oil can be forced to restart is achieved by comparing the plugging strength of the self-assembled particle with the oil restarting pressure in Figure 5. The results are shown below.

Seen from the table (Table 4), the plugging strength in the area with a permeability of 1,265.70 mD is equal to the oil restarting pressure in the area with a permeability of 3.38 mD. That means the oil in the reservoir with a permeability above 3.38 mD can be displaced completely by water if the water channel with a permeability of 1,265.70 mD is plugged by the particle. Thus, combined with the statistical analysis of the permeability in the Fuyu oilfield (Figure 2), most of the remaining oil can be displaced. Similarly, if the self-assembled particle plugs the water channel with a permeability of 3,014.70 mD, the plugging strength can force the oil to be displaced in the areas with permeability over 121.65 mD. So the remaining oil where the permeability is below 121.65 mD cannot be exploited.

**TABLE 4:** Comparison of plugging strength and oil restarting pressure.

Pressure gradient MPa/m	Permeability/mD	
	Particle plugging	Oil restarting
14.05	1,265.70	3.83
0.64	3,014.70	121.65

As the frequency of permeability over 1,500 mD is very low, the water channel in the Fuyu oilfield exists mostly in the areas with a permeability between 1,000 mD to 1,500 mD. Therefore, the self-assembled particle with a diameter of 20–40 μm is suitable for profile control in the Fuyu oilfield.

Furthermore, seen from the research of Dong et al., 2016, the plugging strength of a normal particle in the core with a permeability of 3014.70 mD is only 0.12 MPa/0.3 m (equals to 0.4 MPa/m), which is less than the plugging strength caused by the self-assembled particle injection in the same permeability core (Table 5). The property of self-assembly gives the particle a higher plugging ability and a larger matching window. To sum up, the self-assembled particle is more suitable for profile control than the normal particle.

**TABLE 5:** Plugging strength of self-assembled particle and normal particle.

Permeability mD	Plugging strength/MPa/m	
	Self-assembled particle	Normal particle
3,014.70	0.64	0.40

## Conclusions

1) The permeability in the Fuyu oilfield is normally distributed, and the water channel mostly exists at the areas with a permeability ranging from 1,000 mD to 1,500 mD.
2) The oil restarting pressure increases with the decreasing of permeability, and the increasing rate grows rapidly when the permeability drops below 50 mD.
3) The self-assembled particle with the diameter of 20–40 μm has a good plugging ability in the reservoir with a permeability of 1,265.7 mD, and it can make the remaining oil in the areas with a permeability over 3.38 mD displace by subsequent water flooding.
4) The property of self-assembly can provide the particle with a higher plugging ability and a larger matching window with the water channel than the normal particle. It is suitable for profile control in the Fuyu oilfield.

## Data Availability

The original contributions presented in the study are included in the article/Supplementary Material, further inquiries can be directed to the corresponding author.

## Nomenclature

*L*: Length of the parallel capillary, m
*v*: Moving velocity of oil-water interface, m/d
*x*: Distance between oil-water interface and entrance, m
*r*: Radius of capillary, m
*r*_1_: Radius of smaller-sized capillary, m
*r*_2_: Radius of bigger-sized capillary, m
*p*_A_: Pressures at interface-A, MPa
*p*_B_: Pressures at interface-B, MPa
*μ*_w_: Viscosity of water, mPa.s: Viscosity of oil, mPa.s
*μ*_w_: Viscosity of water, mPa.s: Viscosity of oil, mPa.s
*t*: Time of water flooding, s
*x*_1_: Position of oil-water interface in the smaller size capillary when the oil-water interface in the bigger-sized capillary is arriving at Interface-B, m
*L*_R_: Length of remaining oil in the smaller-sized capillary, m
*S*_o_: Saturation of remaining oil in the smaller-sized capillary, %
