# Supplementary material for: Experimental Investigation of Self-Assembled Particles on Profile Control in the Fuyu Oilfield
Source: Front Chem. 2021 Aug 5;9:681846. doi: 10.3389/fchem.2021.681846 (PMC8375262; doi:10.3389/fchem.2021.681846)
Supplement: Supplementary file 1 [file DataSheet1.docx]

**APPENDIX A:**

**Remaining oil model in heterogeneous reservoir**

Assume the length of the parallel capillary is “L”. The oil is filled and water is injected to displace the oil. The velocity of two-phase flow in single capillary can be expressed as:

$\text{v}=\frac{\text{dx}}{\text{dt}}=\frac{r^{2}(p_{A}-p_{B})}{8[\mu_{w}x+\mu_{o}(L-x)]}$ (A1)

Where “v” is the velocity of oil-water interface; “x” is the distance between oil-water interface and entrance (Interface-A); “r” is the radius of capillary; “μ_w_”, “μ_o_” represent the viscosities of water and oil respectively; “p_A_”, “p_B_” are the pressures at interface-A and Interface-B (**Figure A1**).

Lower permeability area

Higher permeability area

*x*

L

B

A

*q_2_*

*q_w_*

*q_o_*

*p_2_*

*p_A_*

*p_B_*

*r_2_*

*r_1_*

*q_1_*

*p_1_*

Figure A1 The simplified model of micro heterogeneity in reservoir

The integral form can be shown as:

$\int_{0}^{\text{t}} \text{dt}=\frac{8}{r^{2}(p_{A}-p_{B})}\int_{0}^{\text{x}} [\mu_{w}x+\mu_{o}(L-x)]\text{dx}$ (A2)

When injecting water injection for the time of “t”, the oil-water interface moves to the position of “x”,

$\text{t}=\frac{8}{r^{2}(p_{A}-p_{B})}[\mu_{o}L\text{x}+\frac{1}{2}(\mu_{w}-\mu_{o})\text{x}^{2}$ (A3)

$\text{x}=\frac{1}{\mu_{o}-\mu_{w}}\text{[}\mu_{o}L-\sqrt{\mu_{o}^{2}L^{2}-\frac{\text{r}^{2}}{4}(\mu_{o}-\mu_{w})(p_{A}-p_{B})\text{t}}]$ (A4)

After displacing for a period of time, the oil-water interface in the smaller size capillary moves to “x_1_”, while the one in the bigger size capillary is “x_2_”. So the velocities of oil-water interfaces can be described as follow based on Equation (A1).

$\text{v}_{1}\left|_{\text{x}=x_{1}}\right.=\frac{r_{1}^{2}(p_{A}-p_{B})}{8[\mu_{o}L+(\mu_{w}-\mu_{o})x_{1}]}$ (A5)

$\text{v}_{2}\left|_{\text{x}=x_{2}}\right.=\frac{r_{2}^{2}(p_{A}-p_{B})}{8[\mu_{o}L+(\mu_{w}-\mu_{o})x_{2}]}$ (A6)

As we known,

$r_{1}<r_{2}\text{,}\mu_{w}<\mu_{o}\text{,x}_{1}<x_{2}$ (A7)

So

$\text{v}_{1}\left|_{\text{x}=x_{1}}\right.<\text{v}_{2}\left|_{\text{x}=x_{2}}\right.$ (A8)

It means that the oil-water interface in the bigger size capillary takes less time to arrival at the outlet (Interface-B) than in the smaller size capillary. When the oil-water interface in the bigger size capillary is arrival at the Interface-B (x=L), the time can be calculated based on Equitation (A3):

$\text{t}=\frac{4(\mu_{w}+\mu_{o})L^{2}}{r_{2}^{2}(p_{A}-p_{B})}$ (A9)

Combine Equation (A9) and Equation (A4), the position of oil-water interface in the smaller size capillary at this time can be achieved:

$\text{x}_{1}=\frac{\mu_{o}L}{\mu_{o}-\mu_{w}}\text{\{1-}\sqrt{1-(\frac{r_{1}}{r_{2}})^{2}[1-(\frac{\mu_{w}}{\mu_{o}})^{2}]}-(\frac{\mu_{w}}{\mu_{o}})\}$ (A10)

So the oil will be retained in the smaller size capillary and the length of remaining oil can be expressed below:

$\text{L}_{\text{R}}=\text{L-x}_{1}=\frac{\mu_{o}L}{\mu_{o}-\mu_{w}}\text{\{}\sqrt{1-(\frac{r_{1}}{r_{2}})^{2}[1-(\frac{\mu_{w}}{\mu_{o}})^{2}]}-(\frac{\mu_{w}}{\mu_{o}})\}$ (A11)

And the oil saturation in the smaller size capillary can be shown:

$\text{S}_{\text{o}}=\frac{\text{L}_{\text{R}}}{\text{L}}=\frac{\text{L-x}_{1}}{\text{L}}=\frac{\mu_{o}}{\mu_{o}-\mu_{w}}\text{\{}\sqrt{1-(\frac{r_{1}}{r_{2}})^{2}[1-(\frac{\mu_{w}}{\mu_{o}})^{2}]}-(\frac{\mu_{w}}{\mu_{o}})\}$ (A12)
